# Sex Determination in Immature Sierra Nevada Lizard (*Timon nevadensis*)

**DOI:** 10.3390/ani12162144

**Published:** 2022-08-21

**Authors:** Alessandro Vetere, Michela Ablondi, Enrico Bigliardi, Matteo Rizzi, Francesco Di Ianni

**Affiliations:** Department of Veterinary Science, University of Parma, Strada del Taglio 10, 43126 Parma, Italy

**Keywords:** sex determination, reptiles, radiology, lizards

## Abstract

**Simple Summary:**

Sex determination in reptiles is frequently requested by reptile breeders, and it is a real challenge in reptiles with little or no sexual dimorphism, such as in immature subjects. Twenty-three clinically healthy young Sierra Nevada lizards (*Timon nevadensis*) aged between 4 and 6 months were included for sex determination using two techniques: cloacal probing and contrast radiography. Results showed that contrast radiography may have major sensitivity for sex determination compared to probing. Given the above, this technique could represent a valid and less invasive aid for sexing young lizards.

**Abstract:**

Sex determination has a fundamental role in a captive breeding context, both for commercial reasons and in relation to animal welfare itself. However, this can be particularly difficult, especially in reptiles with little or no sexual dimorphism. Twenty-three clinically healthy young Sierra Nevada lizards (*Timon nevadensis*) were included in this study for sex determination. The first attempt at sexing was carried out by cloacal probing. A small, buttoned probe was inserted very gently into the hemipenal pouches, and the length of the inserted part was evaluated and measured. Subsequently, for each animal, a contrast medium was administered into the cloaca, and radiography was performed within 5 min. Through probing, 11 males and 8 females were recognized. The test was, however, equivocal in four subjects. In contrast radiography, 14 males and 9 females were identified. All the animals were rechecked after 8 months through an ultrasound examination, confirming 15 of the 14 previously male sexed animals based on contrast radiography. All the animals identified as female (*n* = 9) by contrast radiography were confirmed. From these results, it seems that contrast radiography may have major sensitivity in sex determination compared to probing. This technique could represent a valid and less invasive aid for sexing young lizards.

## 1. Introduction

The genus *Timon* currently comprises six species found on three different continents [1]. The Asian species *Timon princeps* and *Timon kurdistanicus* inhabit regions in Turkey, Iran and Iraq; and *Timon tangitanus* and *Timon pater* are found in Morocco, Tunisia and Algeria. *Timon nevadensis* and *Timon lepidus* are restricted to Europe [1]. *Timon nevadensis* are present in the south-eastern part of Spain [1]. Adults can be over 130 mm in snout–vent length (SVL) [2] and they are sexually dimorphic: the male is big compared to the female [2]. Mature males display evident prefermoral pores, whereas in females, these pores are not well developed [3]. Juveniles lack evident sexual dimorphism [2,3,4]. While *Timon lepidus* has most recently been assessed for the IUCN Red List of Threatened Species in 2008 as near threatened (NT), there are no data available for *Timon nevadensis*.

Sex determination is frequently required in reptiles, especially for species with no evident sexual dimorphism [5]. Sex identification in lizards is usually visually performed by evaluating secondary sexual characteristics, such as the presence of well-developed femoral pores in males or the presence of brilliant colors of livery. Knowing the sex of individual subjects is essential for zoological facilities or for private breeders to make reproductive pairs. In species where intersex aggression is frequent, a male to female ratio favoring females is typically desired [6,7]. The male and female reproductive systems have different disorders, and knowing the sex of a single animal is very useful for the diagnosis of diseases associated with the reproductive organs [8,9]. Male lizards have a pair of inverted hemipenes [10] placed inside two pouches at the base of the tail; only one hemipene at a time is used for copulation [10], whereas in females, hemipenes are lacking. During copulation, semen is carried from the urogenital opening in the roof of the female vent cloaca along a groove called “sulcus spermaticus.” Hemipene shape varies among reptile species; sometimes they bear spikes or corneal hooks to adhere better to the cloacal mucosa [11]. However, many species lack evident sexual dimorphism [12]. Different techniques for sexing lizards include the following. In probing, a lubricated, stainless-steel probe of the proper diameter is carefully and gently inserted into the side of the vent of the lizard (or even snake) and then directed towards the tail tip along the interior of the side of the tail. If the lizard is a male, the probe will slip inside the hemipenal pouch for approximately one quarter of the tail length, depending on the species [13]. If the probe advances only a short way to a depth of one to six scales [13,14], the lizard is considered a female, as there are no hemipenes for the probe to advance into [15]. Probing is considered quite invasive and should be carefully performed to avoid tissue damage [12,14]. Manual eversion of the hemipenes involves placing a small amount of pressure at the tail base to evert the hemipenes (in males) out of the hemipenal pouches [16]. This practice may be traumatic if performed with excessive force [12,16]. Ultrasonography is also used in lizards as a useful tool to determine follicular development or for sex determination [17,18]. However, in juvenile animals or in very small specimens, the gonads may be difficult or impossible to visualize [12,14]. In addition, ultrasound examination may be difficult to perform in species with thick scales or in subjects where the gut is filled with gas [12]. Contrast radiography is another useful technique involving the use of contrast media introduced into the hemipenal pouches to highlight the presence of the inverted hemipenes inside [12]. The presence of a certain quantity of cellular debris or fluid inside the precloacal pouches seems to interfere with a proper contrast distribution, leading to a false-negative result [19]. Genotipic sex determination via karyotyping and diagnostic techniques such as computed tomography (CT) scanning are useful tools for sex determination in lizards, but they are still expensive [20]. Regarding CT, the image resolution can be inadequate in very small specimens, leading to inconclusive results [12]. The aim of this study was to compare and evaluate the relative diagnostic sensitivity of two different techniques, probing and contrast radiography, for sex determination in immature specimens of Sierra Nevada lizards (*Timon nevadensis*).

## 2. Materials and Methods

**Figure 1:**
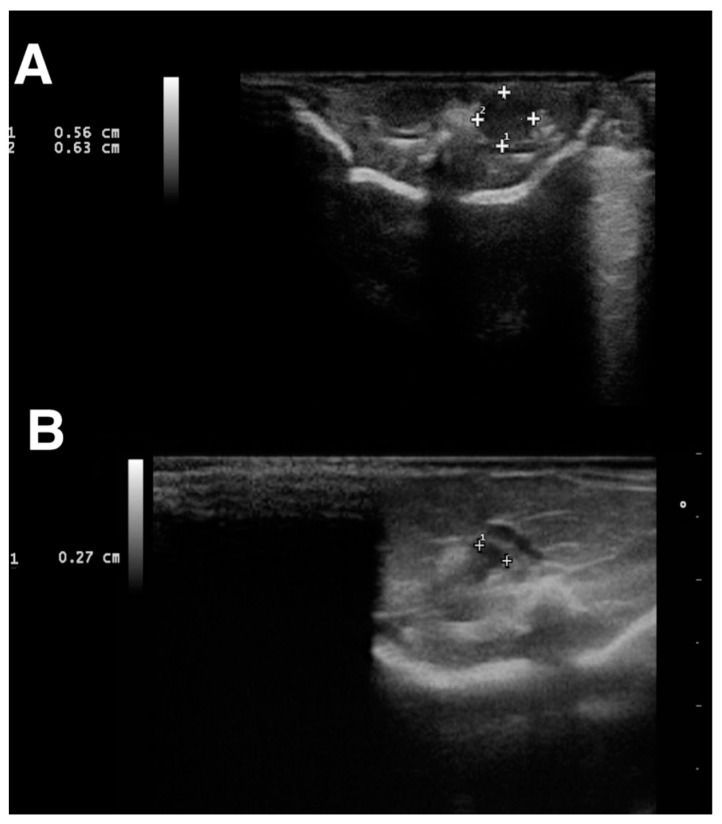
Ultrasonographic examination was performed 8 months after the contrast radiographic study (T3). 1: longitudinal diameter. 2: Transversal diameter (**A**) Transverse plane, dorsal recumbency, dorsocaudal coelom: 0.6 cm c.ca., ovoid in shape and homogeneous in echogenicity organs were identified as testicles in 15 lizards. (**B**) Axial plane, dorsal recumbency, dorsocaudal coelom (left side): ovoid organs characterized by heterogeneous echogenicity due to the presence of numerous 2 mm c.ca anechoic follicles on the organ’s surface were identified as testicles in 8 lizards. 1: transversal diameter.

Ethical approval for the study was given by the University of Parma. The owners gave informed consent to allow participation of their animals in the study. Twenty-three clinically healthy, young Sierra Nevada lizards (*Timon nevadensis*) aged between 4 and 6 months and owned by a private reptile breeder were presented to the Department of Veterinary Science, University of Parma, Italy, for sex determination. The animals weighed, according to an analytical balance (VEVOR^®^ Digital Precision Scale 5000 g × 0.01 g, Taicang Vevor Industry Co., Ltd. 9448 Richmond Pl, #e, Rancho Cucamonga, CA, 91730, USA) from 10 to 18 g (0.022 to 0.039 lb; median, 13.7 g (0.030 lb) and measured from 6 to 12 cm SVL (average 9.8 cm). All the animals were divided individually into different plastic cages and identified with progressive identification numbers. Two different operators performed the imaging, and they were unaware of the sexes of the animals and of the prior results for every performed technique. A first attempt at sex determination was carried out by cloacal probing. The animals were placed under gaseous anesthesia using 2% isoflurane (Isoflo^®^ Zoetis Italia S.r.l, Via Andrea Doria, 41 M, 00,192 Roma RM) for 15 min inside the induction chamber until the rightin reflex was completely lost. A small, buttoned, straight, 1.0 × 60 mm lacrimal duct cannula (E-VET A/S, Ole Rømersvej 26A, 6100 Haderslev, Denmark) was introduced gently into the proctodeum, and then turned backward toward the tail, entering the hemipenal pouch. The length of the inserted part was evaluated and measured. If the probe slipped through the vent inside the hemipenal pouches for more than 5 or 6 caudal scales, the animal was identified as a male. In contrast, if the probe did not go through the vent for more than 4 scales or if any signs of resistance were recorded in both pouches, the animal was classified as female. In every case of dubious results, e.g., different lengths measured for the left and the right pouch in the same subject, the animal was classified as not determined (ND). Subsequently, and blindly, for each animal, 0.1 mL iodinated contrast medium (Ioversol Optiray^®^ 350, Guebert S.p. A, Via Albricci 9, Segrate, Italy) was administered into the postcloacal region by another operator through a buttoned, straight, 1.0 × 60 mm lacrimal duct cannula (E-VET A/S, Ole Rømersvej 26A, 6100 Haderslev, Denmark), and ventro-dorsal (VD) total body radiography was performed within 5 min (Regius 110S, Konica Minolta Health care, Tokyo, Japan). The excess contrast media outlining the cloacal rim was gently cleaned with a piece of a paper towel to avoid artefacts in the radiograms. If one or both of the hemipenes could be visualized, the lizard was classified as male, whereas if the hemipenes were not visualized, the lizard was classified as female. All the animals were rechecked at 8 months (T2) through an ultrasound examination (Esaote Class C, Esaote S.p.a. Via di Caciolle 15, 50127, Florence, Italy) using a 12 MHz linear probe (Esaote Mylab 30 Vet Gold, Esaote S.p.a. Via di Caciolle 15, 50127, Florence, Italy), searching and identifying the gonads. All the animals were placed in dorsal recumbency, and the probe was gently slid cranio-caudally in the third of the coelomic cavity, until the gonads were detected between the spine and the longissimus dorsi in both transversal and longitudinal plane (Figure 1A,B). All the results were compared for statistical analysis.

### Statistical Analysis

**Figure 2:**
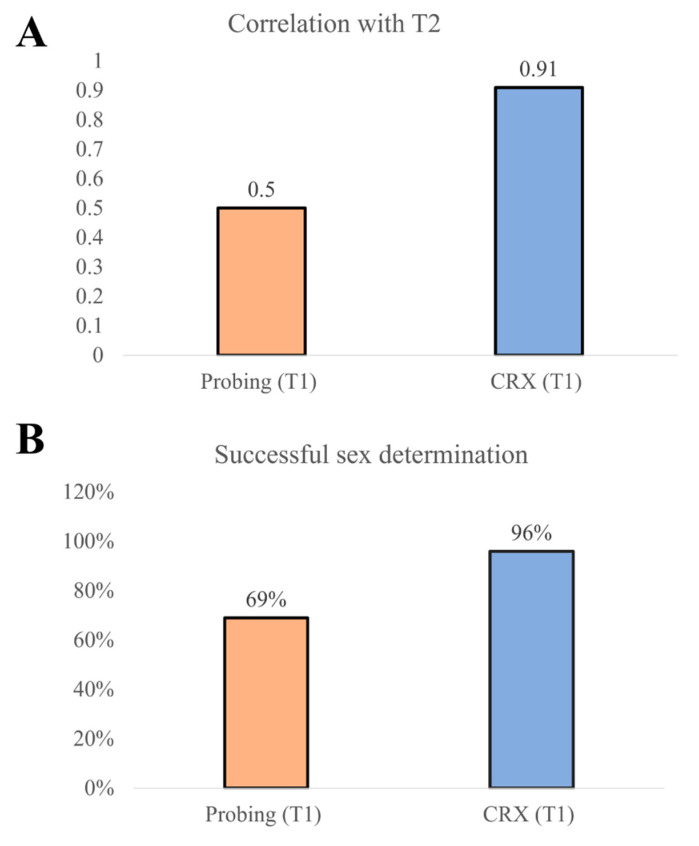
(**A**) A correlation of 0.91 between CRX (T1) and T2 (cor = 0.91, *p* value < 1.592 × 10^−9^) was measured. (**B**) The two-proportion z-test showed that the successful sex determination was 28.1% higher in contrast radiography than in probing.

We obtained a moderate correlation in the case of probing (T1) and T2 (cor = 0.50, *p* value = 0.01475) and a correlation of 0.91 between CRX (T1) and T2 (cor = 0.91, *p* value < 1.592 × 10^−9^) (Figure 2). We used a two-proportion z-test to compare the two observed proportions (sex determination between T1 and CRX T1). The statistical test showed that successful probing sex determination was significantly lower than CRX (*p* value = 0.01) (Figure 2).

## 3. Results

**Figure 3:**
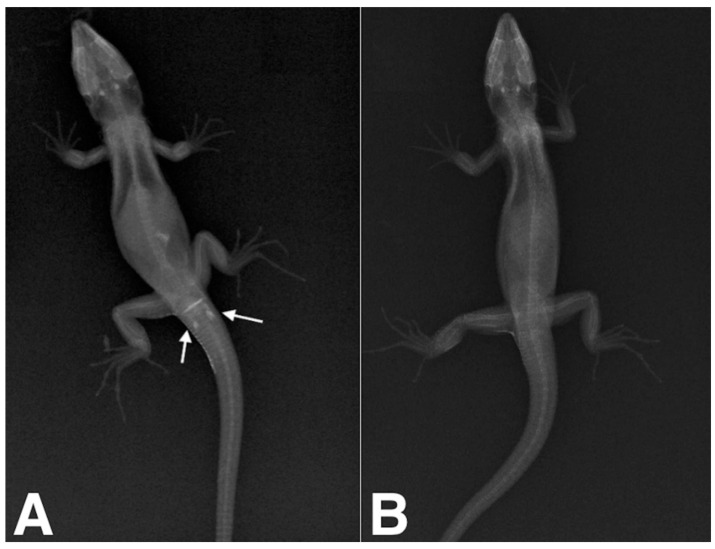
(**A**) Positive contrast radiographic study of a four-month old, 8 cm SVL Sierra Nevada lizard (*Timon nevadensis*); dorsoventral projection. White arrows indicate both the right and left hemipenes. (**B**) Negative contrast radiographic study of a four-month old Sierra Nevada lizard (*Timon nevadensis*); dorsoventral projection. There is no evidence of hemipenes in the postcloacal region.

All the animals maintained spontaneous ventilation during the anesthesia. The lizards were placed individually in a plastic container and monitored during recovery from anesthesia. The animals awakened from 2 to 8 min before the end of the procedure (average 5.2 min), and no adverse effects were noted. Through probing, 11 males and 8 females were recognized. The test was, however, equivocal in four subjects, in which the probe seemed to enter, but opposing an intermediate level of resistance, and different lengths were measured for the left and the right pouch. In contrast radiography, 14 males and 9 females were identified (Table 1). In males, when filled with iodinated contrast medium, hemipenes were seen on radiographs as spindle-shaped cavities pointing caudally to the vent (Figure 3A). In all the females, the contrast did not fill any structures, so no shapes were visible in the X-rays (Figure 3B). For the four subjects for which the test was equivocal (ND), contrast radiography confirmed 1 female and 3 males. After 8 months, ultrasound examination confirmed 14 of the 15 previously male sexed animals based on contrast radiography due to the presence of a 0.6 cm homogeneous echogenicity, ovoid in shape, with organs located in the third caudal of the coelomic cavity (Figure 1A). All the animals identified as female (*n* = 9) by contrast radiography were confirmed due to the pair of ovoid organs characterized by heterogeneous echogenicity due to the presence of numerous, around 2 mm anechoic follicles on the organs’ surfaces (Figure 1B). Only one specimen sexed as a male was revealed to be a female by ultrasound. At 10 and 11 months, three animals died for unknown reasons, and gross necropsy was performed, identifying the gonads and confirming the ultrasonographic sex identification (Table 1), except for one specimen, for which necropsy was not performed due to severe body autolysis. One year after the first measurements (T3), the animals began to show sexual dimorphism: males displayed more developed prefemoral pores than females, and hemipene bulges were evident in all subjects (Table 1).

## 4. Discussion

A variety of techniques have been used for sex determination in reptiles [1,12,13,14,19,21]. Laparoscopy and laparotomy are well described techniques [22,23,24,25,26,27]. While laparotomy for sex determination could be nowadays considered an outdated technique due to the invasiveness of the procedure, laparoscopy, due to the auxilium of a rigid endoscope directly inserted into the coelomic cavity, provides rapid and less invasive gonad visualization in medium size chelonians and lizards [24,25,26]. Probing was performed using a small, metallic, buttoned, straight, 1.0 × 60 mm lacrimal duct cannula. Even if the use of a rigid metallic cannula could be more traumatic than using an endovenous (EV) Teflon catether, we found the rigid cannula easier to introduce inside the cloacal rim without bending it or occluding its tip during contrast media infusion. Contrast radiographies identified at least one hemipene of all the male lizards classified as equivocal with probing. The hemipenes were visualized as a radiopaque elongated structure filled with contrast medium, inverted and directed cranio-caudally (Figure 1a). No adverse effects were seen after the injection of the contrast medium into the cloaca. Although sexual maturity of *Timon* lizards is reached at approximately two years of age [21], the ultrasound examination performed after 8 months (T2) detected and easily identified the gonads [23,24,28]. One animal died at 10 months and two animals at 11 months after the first measurements (T1) for unknown causes. Two lizards were dissected, and even if the cadaveric alterations were moderate to severe, the gonads were recognizable, and the results were consistent with the previously obtained data. One year after the first measurements at T1, the animals were visually rechecked (T3) and began to show signs of sexual dimorphism. In males, the presence of hemipenes was evident by the presence of hemipene bulges. Further research might be needed to confirm the results in a larger sample set. Nevertheless, the two-proportion z-test showed that the successful sex determination was 28.1% higher in contrast radiography compared to probing, which highlights the likely higher sensitivity of the former than the latter.

## 5. Conclusions

From these results, it seems that contrast radiography may have higher sensitivity in sex determination compared to probing. Even if we did not note any procedure-related traumatic complications, we recommend being very gentle during the contrast media injection, because of the thinness of the hemipenal pouches and the very small size of the lizards. Given the above, this technique could represent a valid and less invasive aid for sexing young and very small lizards.

## Figures and Tables

**Table 1:** Identification number (N1 to N23), technique, (probing, contrast radiography, visual check at time T1, T2 and T3), results, (ND = not determinable; M = male; F = female), length (cm) and weight (g) are shown in the table. Three animals died before T3 at 10 (N20) and 11 (N3, N5) months.

No.	Probing (T1)	Contrast Radiography [Crx (T1)]	8 Months Ultrasound Examination (T2)	One Year Visual Check (T3)	Lenght T0, T1 (cm)	Weight (g) (T1)
1	M	M	M	M	10	14
2	ND	M	M	M	9	11
3	ND	F	F	F (Deceased)	9	12
4	F	F	F	F	11	15
5	M	M	M	M (Deceased)	6	10
6	ND	M	M	M	10	14
7	M	M	M	M	9	12
8	F	F	F	F	11	16
9	ND	M	M	M	10	15
10	M	M	M	M	10	16
11	F	F	F	F	11	17
12	F	F	F	F	10	15
13	M	M	M	M	9	11
14	M	M	M	M	9	12
15	M	M	M	M	9	12
16	F	F	M	M	12	18
17	F	F	F	F	11	15
18	M	M	M	M	11	16
19	F	M	M	M	8	12
20	M	M	M	ND (Deceased)	11	15
21	F	F	F	F	9	10
22	M	M	M	M	10	13
23	M	F	F	F	11	15

## Data Availability

Not applicable.

## Abbreviations

CT	computed tomography
EV	endovenous
ND	not determined
NT	near threatened
VD	ventro-dorsal
